# Associations of long-term exposure to air pollution and physical activity with the risk of systemic inflammation-induced multimorbidity in Chinese adults: results from the China multi-ethnic cohort study (CMEC)

**DOI:** 10.1186/s12889-023-17518-2

**Published:** 2023-12-21

**Authors:** Yajie Li, Bing Guo, Qiong Meng, Li Yin, Lin Chen, Xing Wang, Ye Jiang, Jing Wei, Junhua Wang, Jinjie Xia, Zihao Wang, Zhuoma Duoji, Xianzhi Li, Qucuo Nima, Xing Zhao

**Affiliations:** 1 Tibet Center for Disease Control and Prevention, 21 North linkuo Road, Lhasa, Tibet, China; 2 West China School of Public Health and West China Fourth Hospital, Sichuan University, 610041 Chengdu, China; 3 School of Public Health, Kunming Medical University, Kunming, China; 4 Meteorological Medical Research Center, Panzhihua Central Hospital, 617067 Panzhihua, China; 5 Clinical Medical Research Center, Panzhihua Central Hospital, Panzhihua, China; 6 Dali University, Dali, China; 7 Department of Atmospheric and Oceanic Science, Earth System Science Interdisciplinary Center, University of Maryland, College Park, MD USA; 8 School of Public Health, the Key Laboratory of Environmental Pollution Monitoring and Disease Control, Ministry of Education, Guizhou Medical University, Guiyang, China; 9 Chengdu Center for Disease Control and Prevention, Chengdu, China; 10 Chongqing Center for disease Control and prevention, Chongqing, China; 11 Tibet University, Lhasa, China

**Keywords:** Air pollution, Physical activity, Multimorbidity, Systemic inflammation, Particulate matter

## Abstract

**Objective:**

Previous studies proved the effect of long-term exposure to air pollution or physical activity (PA) on the risk of systemic inflammation-induced multimorbidity (SIIM), while the evidence regarding their joint effects was rare, especially in low- and middle-income countries. Therefore, we aimed to examine the extent of interaction or joint relations of PA and air pollution with SIIM.

**Methods:**

This study included 72,172 participants from China Multi-Ethnic Cohort.The average concentrations of ambient particulate matter pollutants (PM_1_, PM_2.5_, and PM_10_) were estimated using satellite-based random forest models. Self-reported information on a range of physical activities related to occupation, housework, commuting, and leisure activities was collected by an interviewer-administered questionnaire. A total of 11 chronic inflammatory systemic diseases were assessed based on self-reported lifetime diagnosis or medical examinations. SIIM was defined as having ≥ 2 chronic diseases related to systemic inflammation. Logistic regression models were used to assess the complex associations of air pollution particulate matter and PA with SIIM.

**Results:**

We found positive associations between long-term air pollution particulates exposure and SIIM, with odds ratios (95%CI) of 1.07 (1.03 to 1.11), 1.18 (1.13 to 1.24), and 1.08 (1.05 to 1.12) per 10 µg/m^3^ increase in PM_1_, PM_2.5_, and PM_10_. No significant multiplicative interaction was found between ambient air pollutant exposure and PA on SIIM, whereas negative additive interaction was observed between long-term exposure to PM_2.5_ and PA on SIIM. The positive associations between low volume PA and SIIM were stronger among those exposed to high-level air pollution particulates. Compared with individuals engaged in high volume PA and exposed to low-level ambient air pollutants, those engaged in low volume PA and exposed to high-level ambient air pollutants had a higher risk of SIIM (OR = 1.49 in PM_1_ exposure, OR = 1.84 in PM_2.5_ exposure, OR = 1.19 in PM_10_ exposure).

**Conclusions:**

Long-term (3 years average) exposure to PM_1_, PM_2.5_, and PM_10_ was associated with an increased risk of SIIM. The associations were modified by PA, highlighting PA’s importance in reducing SIIM for all people, especially those living in high-level air pollution regions.

**Supplementary Information:**

The online version contains supplementary material available at 10.1186/s12889-023-17518-2.

## Introduction

Chronic inflammatory systemic diseases (CIDs), including cardiovascular disease, diabetes, rheumatoid arthritis [1], are the most significant cause of death in the world [2, 3]. Systemic inflammation-induced multimorbidity (SIIM) was defined as having at least two of the CIDs [1, 4]. For several decades the incidence and prevalence of SIIM have been increasing [5], and it is undoubtedly one of the most significant challenges faced by global health care providers [6]. Furthermore, evidence suggests that SIIM related to adverse health outcomes may be enhanced by environmental pollutant exposure [7].

Ambient air pollutants have become top risk factors for global disease burden, causing about 3 million premature deaths every year [8]. Long-term exposure to air pollution may increase the total / differential WBC counts and C-reactive protein level, two markers of systemic inflammation [9]. The particulate matter (PM) induced inflammation has been hypothesized as one of the biological mechanisms linking air pollution and various chronic diseases [10]. Ambient air pollution has adverse effects on various health outcomes, including kidney disease [11], hypertension [12], non-alcoholic fatty liver disease [13], and diabetes [14].

Physical activity (PA) has immediate beneficial effects in reducing the risk of developing and dying from CIDs [15]. However, air pollution may dismiss people from engaging in regular PA. For example, the media alerts of air quality to inform the public about harmful air pollution [16] or the presence of smog [17] may discourage PA behavior. Both air pollution and physical inactivity are positively associated with CIDs [18]. Recently, an increasing number of studies have been conducted to study the joint effects or interaction of PA and air pollution on health outcomes [19, 20]. However, the evidence is mixed. Several studies conducted in low-exposure or medium-exposure settings have shown that there is no interaction between air pollution and PA on long-term health outcomes [9, 21]. A few studies suggest that PA could attenuate the air pollutants-related adverse health effects [22]. The joint effects or interaction of PA and air pollution could have important implications for public health, especially in highly polluted locations.

Although multiple studies have examined the joint effects of PA and long-term air pollution exposure on chronic diseases [19, 20], evidence of SIIM with long-term exposure to ambient air pollution is scarce. To the best of our knowledge, only study conducted in Augsburg found that long-term exposure to NO_2_ and PM_10_ was associated with SIIM among the elderly [23]. Besides, most studies have been conducted in high-income countries, and evidence in low and middle-income countries (LMICs) where air pollution is often more severe is limited [24].

This study aimed to investigate the complex relationships of air pollutants (PM_1_, PM_2.5_, and PM_10_) and PA on SIIM among Chinese adults using baseline data from the China Multi-Ethnic Cohort (CMEC).

## Methods

### Study population

The study populations were derived from the China Multi-Ethnic Cohort study (CMEC). A total of 99,556 participants aged 30–79 years old in Southwestern China were recruited from May 2018 to September 2019 by multistage, stratified cluster sampling method. Data were collected by questionnaires, medical examinations, and clinical laboratory tests. Through face-to-face interviews via electronic questionnaires, the CMEC study elicited information on demographic, socioeconomic, health behaviors, family disease history, physician-diagnosed diseases, indoor air pollution, and other health-related factors. A range of medical examinations, including physical examination, chest radiography, osteopathic examination, and abdominal ultrasonography, were performed to diagnose some physical diseases (e.g., hypertension). In addition, venous blood samples were collected after overnight fasting (at least 8 h) for clinical laboratory tests, that is, blood routine, fasting blood glucose, blood lipid levels, and liver function. More details on the CMEC study have been reported previously [25]. This study was approved by Sichuan University Medical Ethical Review Board (K2016038, K2020022). Written consents from all participants were obtained.

For the current analyses, we excluded [1] those who did not have available residential address information; [2] residents in Aba because they lived nomadically and had no fixed residence; [3] residents in Lhasa because they lived at high altitudes and had a unique dietary habit and thus were less comparable to lowlanders; [4] those who lived at their present address for fewer than three years at the time of the investigation; [5] those with missing information on any outcome, exposure, or adjusted covariates. Ultimately, a total of 72,172 participants were remained in the analyses (Figure S1).

### Exposures assessment

A detailed description of the exposure assessment was described elsewhere [26]. In brief, daily concentrations of 3 air pollutants (PM_1_, PM_2.5_, and PM_10_) were predicted by the space-time extremely randomized trees model using aerosol optical depth, land use information, topographical, and meteorological data [27, 28]. Three-year average concentrations of each air pollutant of participants before the baseline survey were calculated and developed as substitutes for long-term air pollution exposure according to geocoded residential addresses.

The questions on PA were adapted from validated questionnaires used in several other studies [29, 30]. Participants were asked about their usual type and duration of activities related to occupational, chores, traffic, and leisure time exercise during the past year. PA was quantified by metabolic equivalent tasks per day according to the literature [29]. In brief, the number of hours involved in each activity per day was multiplied by the metabolic equivalent for task (MET) score for that activity, and the daily amount of PA was obtained by summing the MET-hours for activities related to occupational, chores, traffic, and leisure time activities. PA and PM are divided into three levels based on their tri-sectional quantiles (1st tertile = low, 2nd tertile = moderate, 3rd tertile = high).

### Outcome assessment

Systemic inflammation-induced multimorbidity (SIIM) including 11 CIDs among the Chinese population [31], was treated as the outcome of our studies. Among these CIDs, definitions of hypertension, diabetes, and metabolic associated fatty liver disease (MAFLD) were based on questionnaires or medical examinations. Seven diagnosed chronic diseases were self-reported (pulmonary heart disease, rheumatic heart disease, coronary heart disease, rheumatic arthritis, stroke, cancer, and rheumatoid arthritis); chronic kidney disease (CKD) was measured by medical examinations. SIIM was defined as having at least two of the defined CIDs [1, 4].

Specific definitions for some CIDs were as follows: [1] hypertension. Participants’ blood pressure was measured three times by using the OMROM HEM-8771 monitor. Hypertension was defined as mean systolic blood pressure ≥ 140 mmHg and/or mean diastolic blood pressure ≥ 90 mmHg and/or has been diagnosed with hypertension by doctors [32] [2]. Diabetes. Fasting plasma glucose (FPG) and glycosylated hemoglobin (HbA1c) were measured enzymatically using the AU5800 Automated Chemistry Analyzer. Diabetes was defined as FPG ≥ 126 mg/dL and/or HbA1c ≥ 6.5% and/or has been diagnosed with diabetes by doctors [33] [3]. MAFLD. According to the definition of MAFLD [34], the MAFLD was diagnosed based on a ultrasonographically confirmed hepatic steatosis plus the presence of any one of the following three metabolic conditions: diabetes mellitus, overweight/obesity, or metabolic dysregulation [4]. CKD. The serum creatinine (SCr) was analyzed using an AU5800 Automated Chemistry Analyzer with the uncompensated Jaffe method involving an alkaline picrate kinetic test. The eGFR level was calculated based on the following MDRD-4 equation: eGFR (mL/min per 1.73 m^2^) = 175 × (SCr)^−1.154^ ×(age)^−0.203^ × 0.742 (for women), where SCr is the serum creatinine level (mg/dL) [35]. Participants with eGFR < 60 mL/min per 1.73 m^2^ were assessed as having CKD according to the KDIGO clinical practice guidelines [36].

### Covariates

Based on the previous literature on air pollution and chronic diseases [37], fully adjusted models included the following other covariates through questionnaires and medical examinations: age (continuous), sex (male or female), marital (cohabited and did not cohabit), ethnic group (Han and minority), region (Yunnan, Guizhou, Chongqing, and Sichuan), annual family income (< 20,000 yuan, 20,000–59,999 yuan, or ≥60,000 yuan), educational level (illiteracy, primary school, junior high school, and high school or above), smoking (never, former, and current), secondary smoking (yes and no), alcohol drinking (never, occasionally, and often), sleep duration (< 6 h per night, 6–8 h per night, and > 8 h per night), indoor air pollution (low, moderate, and high level), body mass index (BMI) (continuous), and dietary pattern (continuous). Indoor air pollution was divided into low, moderate, or high levels according to a summary of cooking behavior, fuel types, and ventilation equipment [26]. BMI was calculated as the body weight (kg) divided by the height squared (m^2^). The dietary pattern was evaluated by the Dietary Approaches to Stop Hypertension (DASH) diet score, which emphasized the consumption of fruits, vegetables, nuts, sodium, low-fat dairy, red and processed meats, and whole-grain intake [38–40].

### Statistical analysis

Differences in essential characteristics between participants with and without SIIM were presented as mean ± standard deviation (SD) and numbers (percentages), and were tested using Student’s *t*-test, the Wilcoxon rank-sum test, and the chi-square test. Pearson correlation test was used to assess the correlation between air pollutants. Logistic regression models were performed to investigate associations of air pollutants (per 10 µg/m^3^ increase) with the risk of SIIM after adjusting potential confounders. Model 1 was adjusted for age, sex, region, ethnic group, marital, annual family income, educational level, smoking, secondary smoking, alcohol drinking, sleep duration, BMI, dietary pattern, and indoor air pollution. Model 2 additionally adjusted for PA and was the main model because it accounted for the most comprehensive covariates. These results are presented as odds ratios (ORs) with 95% confidence intervals (CIs). Given the high correlations among air pollutants, only single-pollutant models were applied.

We conducted a stratified analysis to investigate associations of PA with SIIM in different levels of air pollution subgroups. In this analysis, the reference group was set as the participants engaged in a high volume of PA based on their tri-sectional quantile range of metabolic equivalent tasks per day, and we examined whether low PA was associated with SIIM across different air pollution subgroups.

To quantify the additive and multiplicative interactions, we additionally included a product term of air pollution and PA in the model. The odds ratios (ORs) with 95% confidence intervals (CIs) of the product term were the measure of interaction on the multiplicative scale. We used the relative excess risk due to interaction (RERI) and corresponding 95% confidence intervals (CIs) as the measure of interaction on the additive scale, calculated using the coefficients and corresponding standard errors of the product term, air pollution, and PA, as well as covariance matrix [41]. The basic model was as follows:

*logit* (*p*) = ln ($\frac{P}{1-P}$) = ln (*odds*) = *β*_0_ + *β*_1_A + *β*_2_B + *β*_3_AB

*RERI* = *RR*_A+B+_ - *RR*_A+B−_ - *RR*_A−B+_ +1 = *exp* (*β*_1_ + *β*_2_ + *β*_3_)

– *exp* (*β*_1_) – *exp* (*β*_2_) + 1.

Where *p* denoted the probability of SIIM occurrence, A denoted the air pollution, B denoted the PA, and AB denoted the interaction term between air pollution and PA. The intercept is denoted by *β*_0_, the effect value of air pollution factors is represented by *β*_1_, the effect value of PA is represented by *β*_2_, and the effect value of the interaction term between air pollution and PA is represented by *β*_3_.

According to previous studies, physical inactivity and high levels of air pollution are both associated with a higher risk of chronic diseases [8, 29]. To assess the joint associations, we further classified participants into nine groups according to air pollution (low, moderate, and high) and PA (low, moderate, and high) based on their tri-sectional quantiles and estimated odds ratios of SIIM in different groups compared with those exposures to low-level of air pollution and engaged in a high volume of PA.

We conducted a series of sensitivity analyses. First, we controlled for family disease history (e.g., hypertension, diabetes, cancer, stroke, and acute myocardial infarction) separately in the adjusted models to minimize the influence of hereditary factors. Second, to explore the dose-response relationship between air pollution concentrations and SIIM, restricted splines with three or four degrees of freedom were performed [42]. Third, we used average concentrations of air pollutants for 1, 2, and 4 years before the baseline survey to evaluate the long-term effects of air pollutants exposure.

All of the analyses were performed using R 4.0.2 (R Foundation for Statistical Computing), with a P-value < 0.05 considered statistically significant for a two-tailed test.

## Results

### General characteristics

The average age of the study population was 52.2 years old, and 43,518 participants were women (60.3%). Moreover, 36.3% of the participants were minorities. Approximately half of them had a junior high school or higher education level (51.3%). Participants with SIIM were more likely to be older men, engaged in low PA, with low DASH score, low annual family income, low educational levels, high BMI, current smoking, no cohabitation, often drinking, and short sleep duration. The overall prevalence of SIIM was 21.3% (Table 1 ). The three-year average concentrations of PM_1_, PM_2.5_, and PM_10_ for the overall study were 29.2 µg/m^3^, 40.2 µg/m^3^, and 65.3 µg/m^3^, respectively (Table 2 ).

**Table 1:** Basic characteristics of study participants

Characteristics*	Total (n = 72,172)	Low PA (n = 24,037)	Moderate PA (n = 24,102)	High PA (n = 24,033)	P-value
Age, years (SD)	52.2(11.4)	56.4(12.1)	50.1(11.0)	50.2(9.8)	< 0.001
DASH score (SD)	20.4(4.5)	20.8(4.5)	20.8(4.5)	19.8(4.4)	< 0.001
Physical activity, METs/d (SD)	26.8(18.3)	8.9(4.2)	23.3(4.9)	48.1(13.5)	< 0.001
BMI (SD)	24.0(3.4)	24.3(3.4)	24.0(3.3)	23.8(3.4)	< 0.001
Three-year average PM_1_, µg/m^3^ (SD)	30.4(11.3)	33.2(11.5)	31.4(11.4)	26.6(9.9)	< 0.001
Three-year average PM_2.5_, µg/m^3^ (SD)	38.7(12.4)	41.9(12.1)	39.9(12.3)	34.4(11.7)	< 0.001
Three-year average PM_10_, µg/m^3^ (SD)	65.2(16.2)	69.4(16.1)	66.7(16.1)	59.4(14.7)	< 0.001
Sex, n (%)					< 0.001
Male	28,654(39.7)	9,851(41.0)	9,305(38.6)	9,498(39.5)	
Female	43,518(60.3)	14,186(59.0)	14,797(61.4)	14,535(60.5)	
Ethnic group (%)					< 0.001
Han	46,000(63.7)	16,738(69.6)	15,854(65.8)	13,408(55.8)	
Minority	26,172(36.3)	7,299(30.4)	8,248(34.2)	10,625(44.2)	
Region (%)					< 0.001
Yunnan	20,261(28.1)	4,650(19.3)	5,886(24.4)	9,725(40.5)	
Chongqing	18,943(26.2)	7,095(29.5)	6,971(28.9)	4,877(20.3)	
Guizhou	15,283(21.2)	4,584(19.1)	4,887(20.3)	5,812(24.2)	
Sichuan	17,685(24.5)	7,708(32.1)	6,358(26.4)	3,619(15.1)	
Marital, n (%)					< 0.001
Married/Cohabitating	64,361(89.2)	20,565(85.6)	21,729(90.2)	22,067(91.8)	
Unmarried/divorced/widowed	7,811(10.8)	3,472(14.4)	2,373(9.8)	1,966(8.2)	
Annual family income, yuan/year (%)					< 0.001
<20,000	25,094(34.8)	7,614(31.7)	7,265(30.1)	10,215(42.5)	
20,000–59,999	26,044(36.1)	8,254(34.3)	8,243(34.2)	9,547(39.7)	
≥ 60,000	21,034(29.1)	8,169(34.0)	8,594(35.7)	4,271(17.8)	
Education level, n (%)					< 0.001
Illiteracy	16,657(23.1)	5,271(21.9)	4,864(20.2)	6,522(27.1)	
Primary school	18,447(25.6)	5,794(24.1)	5,112(21.2)	7,541(31.4)	
Junior high school	19,713(27.3)	6,490(27.0)	6,284(26.1)	6,939(28.9)	
High school or higher	17,355(24.0)	6,482(27.0)	7,842(32.5)	3,031(12.6)	
Smoking status, n (%)					< 0.001
Never	53,457(74.1)	17,390(72.3)	18,168(75.4)	17,899(74.5)	
Former	3,685(5.1)	1,646(6.8)	1,079(4.5)	960(4.0)	
Current	15,030(20.8)	5,001(20.8)	4,855(20.1)	5,174(21.5)	
Secondary smoking, n (%)					< 0.001
Yes	37,331(51.7)	11,873(49.4)	12,824(53.2)	12,634(52.6)	
No	34,841(48.3)	12,164(50.6)	11,278(46.8)	11,399(47.4)	
Alcohol drinking status, n (%)					< 0.001
Never	40,155(55.6)	13,614(56.6)	12,733(52.8)	13,808(57.5)	
Occasionally	22,047(30.5)	7,056(29.4)	8,213(34.1)	6,778(28.2)	
Often	9,970(13.8)	3,367(14.0)	3,156(13.1)	3,447(14.3)	
Sleep duration, hours/day (%)					< 0.001
< 6	9,372(13.0)	3,803(15.8)	2,741(11.4)	2,828(11.8)	
6–8	51,448(71.3)	16,578(69.0)	17,887(74.2)	16,983(70.7)	
> 8	11,352(15.7)	3,656(15.2)	3,474(14.4)	4,222(17.6)	
Indoor air pollution, n (%)					< 0.001
Low	11,501(15.9)	4,109(17.1)	3,747(15.5)	3,645(15.2)	
Moderate	57,046(79.0)	19,030(79.2)	19,304(80.1)	18,712(77.9)	
High	3,625(5.0)	898(3.7)	1,051(4.4)	1,676(7.0)	
SIIM					< 0.001
No	56,781(78.7)	17,154(71.4)	19,570(81.2)	20,057(83.5)	
Yes	15,391(21.3)	6,883(28.6)	4,532(18.8)	3,976(16.5)	

BMI indicates body mass index; DASH, dietary approaches to stop hypertension; METs, metabolic equivalent tasks; PM_1_, the particle with an aerodynamic diameter of 1 μm or less; PM_2.5_, the particle with an aerodynamic diameter of 2.5 μm or less; PM_10_, the particle with an aerodynamic diameter of 10 μm or less

*Data are the mean (SD) for continuous variables and number (percentage) for categorical variables

**Table 2:** Three-year average concentrations of ambient air pollutants

Variables	3-year average concentrations						Pearson correlation coefficients			
	Minimum	Maximum	P_25_	P_50_	P_75_	IQR	PM_1_	PM_2.5_	PM_10_	NO_2_
PM_1_	13.0	54.5	19.3	29.2	37.1	17.8	1.00	0.96	0.96	0.90
PM_2.5_	16.2	61.6	25.1	40.2	48.2	23.1	0.96	1.00	0.98	0.85
PM_10_	33.7	100.6	49.7	65.3	75.7	26.0	0.96	0.98	1.00	0.89

PM_1_, the particle with an aerodynamic diameter of 1 μm or less; PM_2.5_, the particle with an aerodynamic diameter of 2.5 μm or less; PM_10_, the particle with an aerodynamic diameter of 10 μm or less

### Associations of ambient air pollutant exposure with SIIM

Table 3 demonstrates a statistically significant association between heightened levels of air pollutants and an increased susceptibility to SIIM, even after accounting for potential confounding factors. For example, for every 10 µg/m^3^ increase in PM_1_, PM_2.5_, and PM_10_, the odds ratios of SIIM were 1.07 (95%CI, 1.03–1.11), 1.18 (95%CI, 1.13–1.24), and 1.08 (95%CI, 1.05–1.12), respectively. The results remained similar after adjusting for various family disease histories (Table S1).

**Table 3:** ORs and 95% CI for systemic inflammation-induced multimorbidity associated with per 10-µg/m^3^ increase in ambient air pollutants

Pollutant	Model 1	Model 2
PM_1_	1.08 (1.05–1.12)	1.07 (1.03–1.11)
PM_2.5_	1.20 (1.15–1.26)	1.18 (1.13–1.24)
PM_10_	1.10 (1.06–1.13)	1.08 (1.05–1.12)

Model 1 was adjusted for age, sex, marital, ethnic group, region, annual family income, educational level, smoking, secondary smoking, alcohol drinking, sleep duration, dietary pattern, indoor air pollution, and BMI. Model 2 was further adjusted for physical activity

PM_1_, the particle with an aerodynamic diameter of 1 μm or less; PM_2.5_, the particle with an aerodynamic diameter of 2.5 μm or less; PM_10_, the particle with an aerodynamic diameter of 10 μm or less

According to the findings presented in Figure S2, the restricted spline regressions indicate that the relationships between SIIM and PM exposure appear to follow a linear trend in the adjusted model. Table S2 indicated similar effect estimates for SIIM when average ambient air pollution concentrations from various years prior to the survey were utilized as the exposure variable. Specifically, increments of 10 µg/m^3^ in PM_1_ over the average concentration of four years were linked to increases in the odds ratio for SIIM. The minimal disparity in outcomes across the four-year period demonstrated the reliability of the findings.

### Interaction and joint analysis between PM exposure and PA and SIIM

**Fig. 1:**
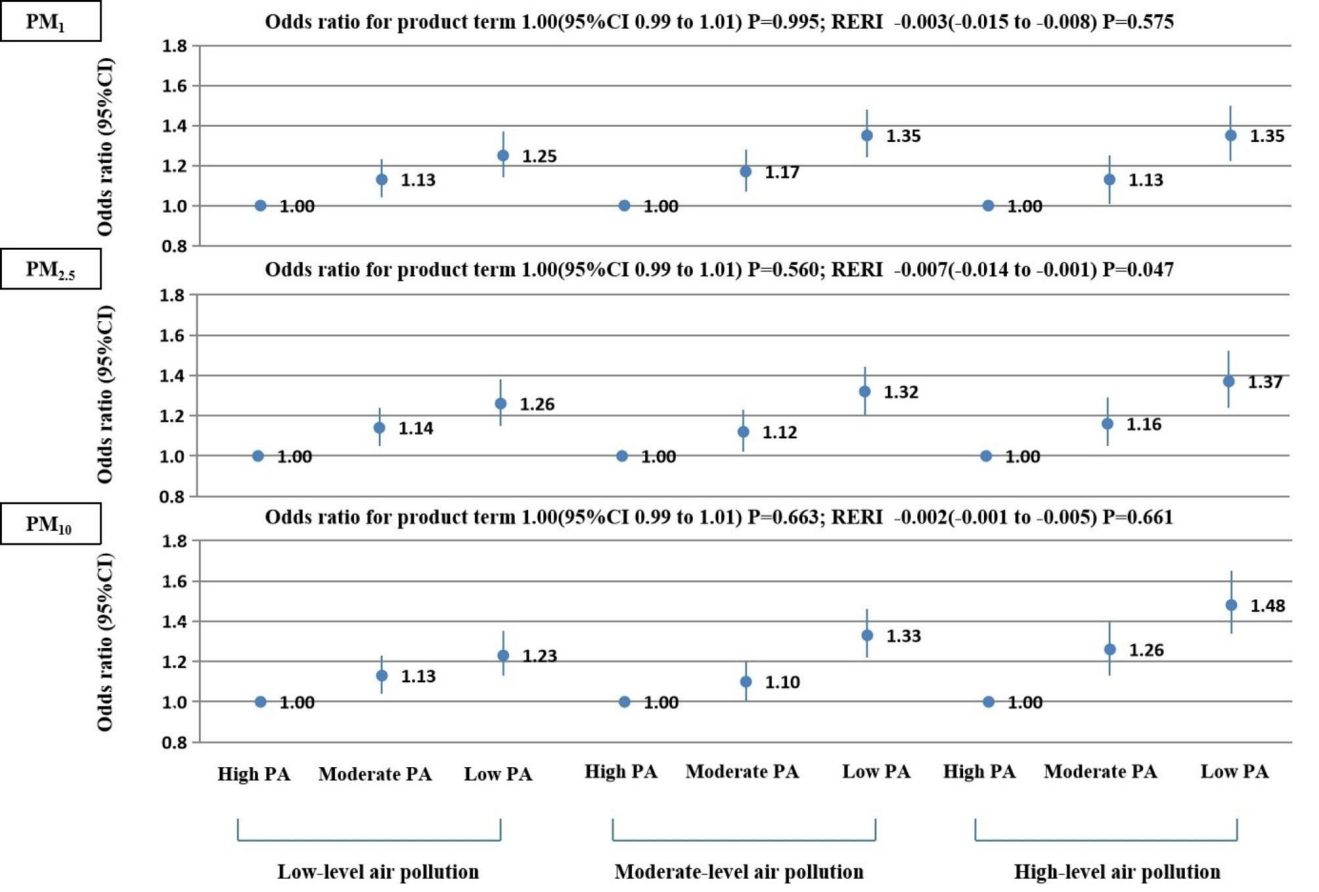
Associations of physical activity with systemic inflammation-induced multimorbidity by levels of air pollution. Odds ratio were adjusted for age, sex, marital, ethnic group, region, annual family income, educational level, smoking, secondary smoking, alcohol drinking, sleep duration, dietary pattern, indoor air pollution, and BMI. PM_1_, the particle with an aerodynamic diameter of 1 μm or less; PM_2.5_, the particle with an aerodynamic diameter of 2.5 μm or less; PM_10_, the particle with aerodynamic diameter of 10 μm or less

The relative excess risk due to interaction of PM exposure and PA on SIIM was presented by RERI. No significant multiplicative interaction was found between PM exposure and PA on SIIM, whereas a negative additive interaction was observed between PM_2.5_ exposure and PA on SIIM (*P* for interaction < 0.05) (Fig. 1).

Low volume PA was associated with higher risks of SIIM among individuals exposed to various subgroups of ambient air pollutants, whereas the associations were stronger among those exposed to a high ambient air pollutant subgroup (Fig. 1). For example, the odds ratio for those engaged in low volume PA compared with high volume PA for SIIM were 1.26 (1.15–1.38) among individuals exposed to low-level of PM_2.5_, 1.32 (1.20–1.44) among those exposed to Moderate-level of PM_2.5_, and 1.37 (1.24–1.52) among those exposed to high-level of PM_2.5_. Similar patterns were found for PM_1_ and PM_10_.

**Fig. 2:**
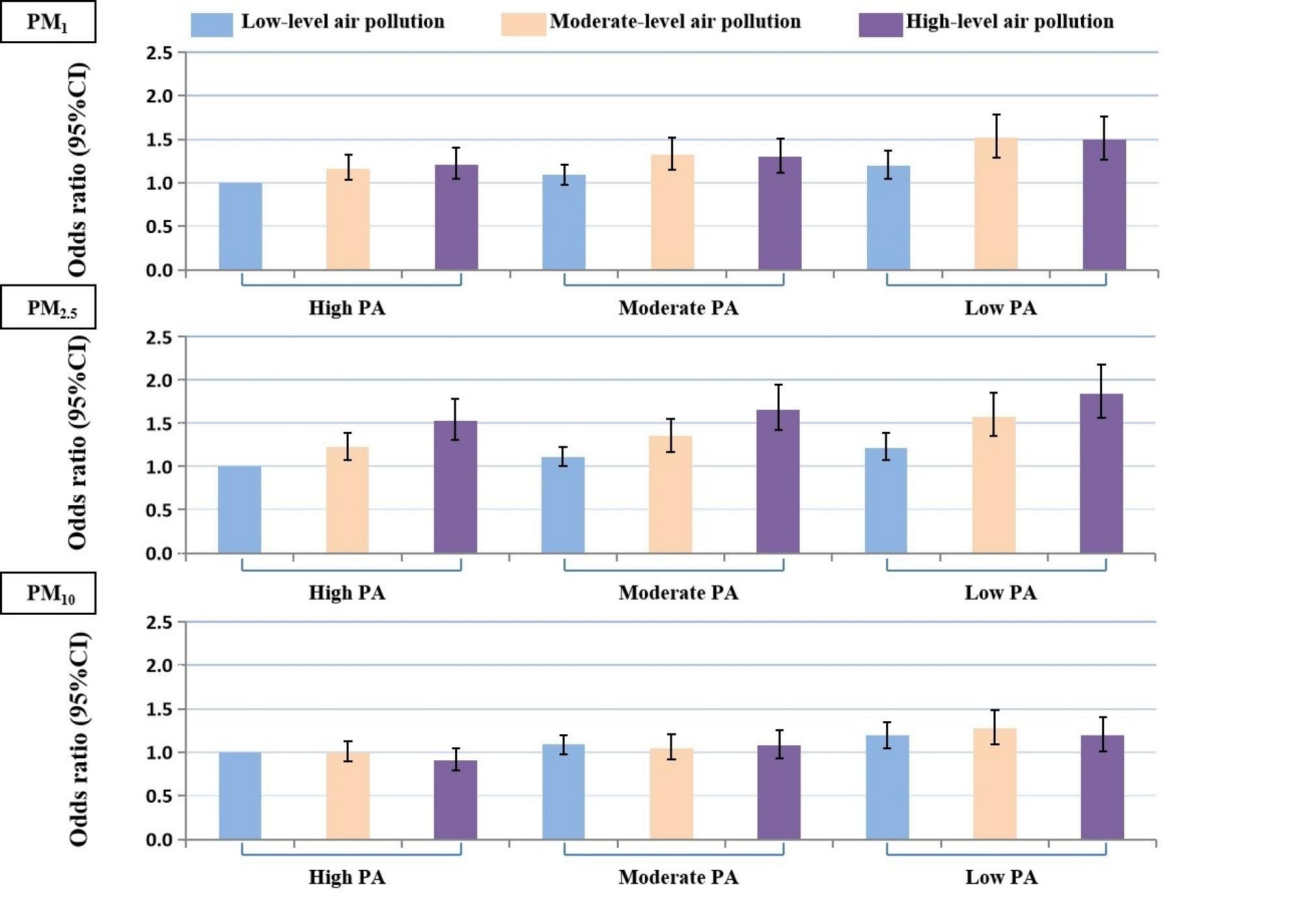
**Joint associations of long-term exposure to air pollution and physical activity with systemic inflammation-induced multimorbidity**. Odds ratio were adjusted for age, sex, marital, ethnic group, region, annual family income, educational level, smoking, secondary smoking, alcohol drinking, sleep duration, dietary pattern, indoor air pollution, and BMI. PM_1_, the particle with an aerodynamic diameter of 1 μm or less; PM_2.5_, the particle with an aerodynamic diameter of 2.5 μm or less; PM_10_, the particle with an aerodynamic diameter of 10 μm or less

Figure 2 shows the joint association of ambient air pollutant exposure and PA on SIIM and the odds ratio for individuals engaged in low volume PA and exposed to high-level ambient air pollutants compared with those engaged in high volume PA and exposed to low-level ambient air pollutants for SIIM were 1.49 (1.27–1.76) in PM_1_ exposure, 1.84 (1.56–2.18) in PM_2.5_ exposure, and 1.19 (1.01–1.40) in PM_10_ exposure.

## Discussion

### Main findings

To our knowledge, this is the first study to examine the associations of SIIM with long-term air pollution particulates exposure and PA among adults aged 30 to 79 years. Our study found that long-term (3 years average) air pollution particulates exposure was positively associated with SIIM. A significant additive interaction was found between long-term ambient PM_2.5_ exposure and PA on SIIM, and the associations between PA and SIIM were stronger among those exposed to high-level air pollution particulates. In addition, the highest risks of SIIM were seen in individuals engaged in low volume PA and exposed to high or moderate levels of air pollution particulates. The results showed that some measures need to be taken to solve the problems of physical inactivity and ambient air pollution, which could contribute to reduce the burden of SIIM.

### Potential mechanism

Although the underlining mechanisms of the association between long-term air pollution particulates exposure and SIIM remain largely unknown, several possible pathways have been suggested. First, air pollutants may lead to the generation of endogenous pro-inflammatory mediators, oxidative stress, autonomic nervous system imbalance, endothelial dysfunction, and plasma viscosity increases [43, 44], resulting in SIIM. Second, air pollution exposure may be associated with abnormal methylation levels of global DNA and specific genes involved in blood pressure regulation, glucose-homeostasis, and lipid metabolism pathways [45]. Third, severe particulate air pollution can restrict people from engaging in PA [46], which may lead to obesity. Obesity has been acknowledged as a risk factor for the development of most CIDs [47].

The biological mechanism of the interaction and joint effects of PA and air pollution on SIIM might be related to low-grade systemic inflammation. Long-term air pollution particulates exposure has adverse effects on multimorbidity by inducing systemic inflammatory processes [48, 49]. However, one of the key mechanisms by which PA exerts beneficial health effects appears to be due to its capacity to reduce chronic low-grade inflammation [50]. Skeletal muscle is an endocrine organ that produces a variety of metabolic factors, which provides a mechanical link between muscle contraction and its beneficial effects on systemic inflammation and health [51]. In addition, PA may increase the inhalation of air pollutants due to higher ventilation, which could amplify the adverse health effects of air pollutants [9]. Furthermore, ambient air pollution could create a barrier for doing outdoor PA, and that the health risk from exposure to air pollution could weaken the benefits of PA.

### Comparison with other studies

Our findings indicated long-term exposures to PM_1_, PM_2.5_, and PM_10_ were all positively associated with an increased risk of SIIM. This was consistent with a previous analysis in the KORA-Age study, which also found that long-term exposure to NO_2_ and PM_10_ were associated with SIIM among the elderly [23]. In addition, there is evidence that shows that extended exposure to air pollution could elicit health risks during multimorbidity clinic visits [52]. Nevertheless, research on the relationship between long-term air pollution particulates exposure and multimorbidity is relatively scarce. There is an urgent need for further studies to be conducted in different regions.

In our study, we confirmed that low PA was associated with higher risks of SIIM, regardless of air pollution particulates exposure. The results were consistent with some previous studies [53, 54]. In addition, negative additive interaction was found between long-term ambient PM_2.5_ exposure and PA on SIIM. The evidence on the interaction between long-term ambient air pollutant exposure and PA on health effects is mixed. Several studies suggest that there is no interaction between air pollution and PA on health outcomes for systemic inflammation and chronic obstructive pulmonary disease [9, 20]. A few studies suggest that PA may have a beneficial effect in protecting against adverse effects of air pollution on blood pressure [55]. The exact reasons for the inconsistent findings were unclear but might be partly due to different levels of air pollution settings [18]. Nevertheless, our finding indicates that participants can benefit from PA despite inhaling a large amount of PM_2.5_ during PA, which could reduce the public’s doubts about the hazards of exposure to air pollution during PA. More studies are still needed to understand the complex relations between air pollution particulates exposure and PA on SIIM in different regions with different air pollution levels.

The associations of PA with SIIM were stronger among those exposed to high-level of air pollution particulates exposure, which highlighted the necessity of PA modification, especially among those living in high-level air pollution particulates exposure regions. Also, there were strong indications that estimated effects of high-level ambient air pollutants with low PA on SIIM were larger compared with those of low-level ambient air pollutants with high PA. These findings had important public health implications in identifying subgroups (e.g., participants exposed to high-level of ambient air pollutants with low PA) that would benefit most from the intervention.

### Strengths of this study

The main strength of this study was to examine associations of long-term air pollution particulates exposure and PA with SIIM using a large sample size in China. The large sample size allowed us to perform the joint and stratified analyses with sufficient statistical power. In addition, the wide concentration range of air pollutants in our study has implications for both high and low levels of pollution areas.

### Limitations of this study

However, there were limitations to this current study. First, since the time period for PM exposure is assessed for a three-year average value, those who had any events of SIIM within or before the three-year exposure measurement period should be excluded from the analysis. However, since our study design is cross-sectional, it may lead to an inversion of cause and effect. Second, the SIIM in our study included only 11 chronic diseases related to systemic inflammation, and most of the diseases were self-reported. Third, the definition of multimorbidity was simply to count the number of chronic diseases without accounting for the different clusters and severity of chronic diseases. Fourth, the information about PA, history of disease, and sleep duration was self-reported by participants, which may lead to some recall bias. Finally, there may be other confounding variables that we did not control for due to limited data availability.

### Perspectives

Long-term exposure to PM_1_, PM_2.5_, and PM_10_ were positively associated with an increased risk of SIIM. Moreover, there was a significant additive interaction between long-term ambient PM_2.5_ exposure and PA on SIIM. Individuals engaged in low PA and exposed to high or moderate level of air pollution particulates had the highest risks of SIIM, which highlights the importance of PA modification in reducing SIIM for all people, especially those living in high-level air pollution regions.

### Electronic supplementary material

Below is the link to the electronic supplementary material.

Supplementary Material 1

## Data Availability

The data sets generated and analysed during the current study are not publicly available due property rights protection but are available from the corresponding author on reasonable request.

## Abbreviations

SIIM: Systemic inflammation-induced multimorbidity
PA: Physical activity
CMEC: The China Multi-Ethnic Cohort Study
CIDs: Chronic inflammatory systemic diseases
PM: Particulate matter
LMICs: Low and middle-income countries
OR: Odds ratio
CI: Confidence interval
BMI: Body mass index
DASH: Dietary Approaches to Stop Hypertension
MAFLD: Metabolic associated fatty liver disease
CKD: Chronic kidney disease
FPG: Fasting plasma glucose
HbA1c: Glycosylated haemoglobin
MET: Metabolic equivalent for task
