# Supplementary material for: Associations of long-term exposure to air pollution and physical activity with the risk of systemic inflammation-induced multimorbidity in Chinese adults: results from the China multi-ethnic cohort study (CMEC)
Source: BMC Public Health. 2023 Dec 21;23:2556. doi: 10.1186/s12889-023-17518-2 (PMC10734128; doi:10.1186/s12889-023-17518-2)
Supplement: Supplementary file 1 — Supplementary Material 1 [file 12889_2023_17518_MOESM1_ESM.docx]

**Supplementary material**

**Table S1 The associations of systemic inflammation-induced multimorbidity with each 10-μg/m^3^ increase in ambient air pollutants**

| Pollutant | Model a | Model b | Model c | Model d | Model e |
| --- | --- | --- | --- | --- | --- |
| PM_1_ | 1.07 (1.03-1.11) | 1.07 (1.03-1.11) | 1.07 (1.03-1.11) | 1.07 (1.04-1.11) | 1.07 (1.03-1.11) |
| PM_2.5_ | 1.17 (1.12-1.23) | 1.18 (1.12-1.24) | 1.18 (1.13-1.24) | 1.18 (1.13-1.24) | 1.18 (1.13-1.24) |
| PM_10_ | 1.08 (1.04-1.11) | 1.08 (1.05-1.12) | 1.08 (1.05-1.12) | 1.09 (1.05-1.12) | 1.08 (1.05-1.12) |

Model a-e was additionally adjusted for family disease history of diabetes, hypertension, cancer, stroke, and acute myocardial infarction.

PM_1_, the particle with an aerodynamic diameter of 1 μm or less; PM_2.5_, the particle with an aerodynamic diameter of 2.5 μm or less; PM_10_, the particle with an aerodynamic diameter of 10 μm or less.

Figure **S**1 Flow charts for participants enrollment


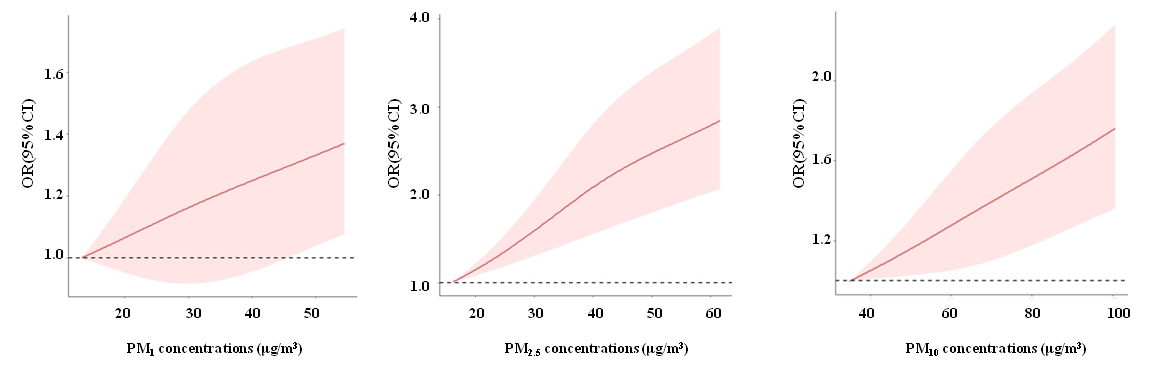


**Figure S2. OR and 95%CI for the risk of systemic inflammation-induced multimorbidity along with the changes of air pollutants from the restricted splines regression model**

Models were adjusted for age, sex, marital, ethnic group, region, annual family income, educational level, smoking, secondary smoking, alcohol drinking, sleep duration, physical activity, dietary pattern, indoor air pollution, and BMI.

Abbreviations: PM_1_, the particle with an aerodynamic diameter of 1 μm or less; PM_2.5_, the particle with an aerodynamic diameter of 2.5 μm or less; PM_10_, the particle with an aerodynamic diameter of 10 μm or less.

**Table S2 Association of systemic inflammation-induced multimorbidity with per 10-μg/m^3^ increase of ambient air pollution in different exposure times**

| Ambient particulate exposure times | OR(95%CI) | | |
| --- | --- | --- | --- |
|  | PM_1_ | PM_2.5_ | PM_10_ |
| One-year | 1.06 (1.01-1.10) | 1.13 (1.07-1.20) | 1.06 (1.02-1.09) |
| Two-year | 1.07 (1.03-1.11) | 1.19 (1.13-1.25) | 1.08 (1.05-1.12) |
| Four-year | 1.08 (1.04-1.12) | 1.20 (1.14-1.26) | 1.09 (1.06-1.13) |

Note: Odds ratio were adjusted for age, sex, marital, ethnic group, region, annual family income, educational level, smoking, secondary smoking, alcohol drinking, sleep duration, dietary pattern, indoor air pollution, physical activity and BMI.

PM_1_, the particle with an aerodynamic diameter of 1 μm or less; PM_2.5_, the particle with an aerodynamic diameter of 2.5 μm or less; PM_10_, the particle with an aerodynamic diameter of 10 μm or less.
